# High fish density delays wound healing in Atlantic salmon (*Salmo salar*)

**DOI:** 10.1038/s41598-018-35002-5

**Published:** 2018-11-15

**Authors:** Lene Rydal Sveen, Gerrit Timmerhaus, Aleksei Krasnov, Harald Takle, Sigurd Olav Stefansson, Sigurd Olav Handeland, Elisabeth Ytteborg

**Affiliations:** 1 University of Bergen, Postboks 7800, 5020 Bergen, Norway; 2 Nofima, Osloveien 1, 1430 Ås, Norway; 3 Uni Research, Thormøhlens Gate 55, 5008 Bergen, Norway; 4 Cermaq Group AS, Dronning Eufemias gate 16,0102, Oslo, Norway

## Abstract

In this study, we look closer at how high fish densities influence wound repair mechanisms in post-smolt Atlantic salmon. The fish were wounded with a 5 mm skin punch biopsy needle and stocked at two different densities, a high fish density (100 kg/m^3^) treatment and a low fish density treatment (20 kg/m^3^) serving as the control. The healing wounds were followed for 57 days with samples taken 1, 3, 7, 14, 36, 43 and 57 days post wounding. The transcriptomic analysis suggests that high fish density enhance inflammation and represses cell proliferation, tissue secretion and collagen synthesis in the healing wounds. The histological analysis further showed delayed epidermal and dermal repair in the high fish density treatment compared to control. The overall wound contraction was also altered by the treatment. In conclusion, high fish density enhances immune responses and delay tissue repair, which ultimately results in delayed wound healing.

## Introduction

Sustainable growth in the Atlantic salmon (*Salmo salar*) aquaculture sector depends on good fish health and welfare. Currently, low-cost open sea cages are predominantly used for on-growth of salmon. However, there are concerns related to salmon lice (*Lepeophtheirus salmonis*), escapees, nutrient discharge and fish mortalities^1^. Development of semi-closed containment technologies (S-CCS) in sea and closed containment systems (CCS) in land-based facilities are promising strategies aiming to solve these problems, and provide further expansion of the Atlantic salmon production in Norway^2^. CCS and S-CCS are mainly intended for the production of post-smolt during a limited period after seawater transfer. As of to date, no regulation exist for maximum fish density in CCS^3^. In contrast, the maximum allowed fish density in sea cages are 25 kg/m^3^.

Crowding and high fish densities may weaken the skin and increase the risk of mechanical damage^4–8^. Damage to the skin may threaten the barrier function of the fish resulting in reduced animal welfare^9^. If the skin is severely wounded (epidermal and dermal damage), a well-conserved wound healing cascade is activated in order to restore tissue integrity. The wound healing cascade is initiated by the re-epithelialization processes, accompanied by inflammation and later onset of tissue repair and remodeling^10^. In salmonids, re-epithelialization is initiated immediately by wounding^11,12^, while inflammation lasts more than two weeks, accompanied by tissue repair which may be active more than 100 days post wounding^13^.

Cutaneous diseases and wounds are common for many farmed fish species^14,15^. Hence, there has been a few studies reporting effects of environmental factors, hormones and dietary components on the healing rate of deep cutaneous wounds. In Atlantic salmon, low water temperature result in delayed epidermal repair^11,16^, while the stress hormone cortisol delay the dermal repair processes^12^. In contrast, dietary intake of zinc enhanced epidermal repair in Atlantic salmon^16^, while the dermal repair was promoted in Rainbow trout (*Oncorhynchus mykiss*) with high dietary levels of vitamin C^17^. Other factors such as therapeutics and immunostimulants may also affect the wound healing rate in fish^18–20^. To the best of our knowledge, there is no study investigating the relationship between densities and deep cutaneous wound healing in fish.

The present study was designed to test the hypothesis that high fish density delays wound repair in post-smolt Atlantic salmon. The fish were wounded with a 5 mm biopsy needle and stocked at two different densities, a high fish density treatment (HFD) (100 kg/m^3^) and a low fish density treatment (20 kg/m^3^) serving as control. A 15k oligonucleotide array was used to observe changes in gene transcripts, while histology and photography were used to assess changes in wound morphology and contraction. Overall, our results show that HFD induces prolonged activation of inflammation and transient repression of tissue repair, which results in alterations in wound contraction.

## Results and Discussion

### Cortisol levels

**Figure 1:**
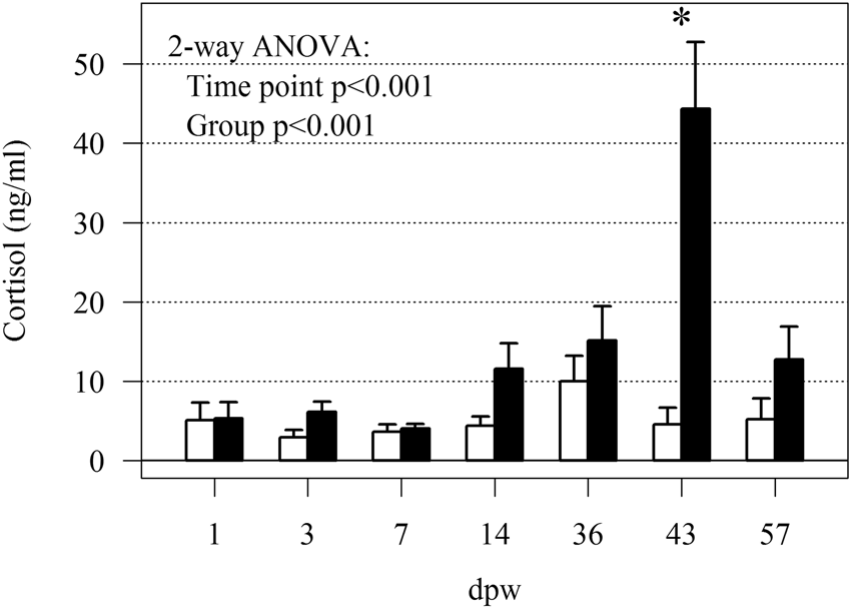
Plasma cortisol response to the treatment. The bars show mean plasma cortisol levels and error bars SEM, HFD (black bars) and control (white bars). A significant difference in plasma cortisol levels between time point and treatment was indicated by 2-way ANOVA (p < 0.001). Differences between groups (for each time point, Tukey *post-hoc* test) are indicated with a star (p < 0.05). N = 12 for treatment and time point.

The HFD treatment did not affect mortality. The overall mortality rate was low, <1% in the control and <5% in the HFD treatment. Similarly, Calabrese *et al*. 2017 did not observe increased mortality in fish reared at high densities (25–125 kg/m^3^). Significant differences in plasma cortisol levels (2-way ANOVA, p < 0.001) were found both between time points and treatment (Fig. 1). However, the post-hoc analysis only showed significant differences between groups at 43 days post wounding (dpw). Similar observations were done in our previous experiment investigating animal welfare and the effect of five different fish densities (25, 50, 75, 100 and 125 kg/m^3^)^4,5^. In this previous study, plasma cortisol levels peaked in the intermediate density treatment (75 kg/m^3^) after four weeks, whereas plasma cortisol levels in the highest density treatment (125 kg/m^3^) peaked after eight weeks. Other studies have also demonstrated that fish exposed to crowding have limited cortisol response. Basal levels of plasma cortisol in unstressed salmonid fish are normally in the range 0–5 ng/mL, while crowding resulted in an elevation of plasma cortisol to 10 mg/mL^21^. These results suggest that cortisol as a sole indicator of animal performance during long-term intensive rearing conditions may be misleading and other parameters should be included in order to assess animal welfare.

### Wound contraction

**Figure 2:**
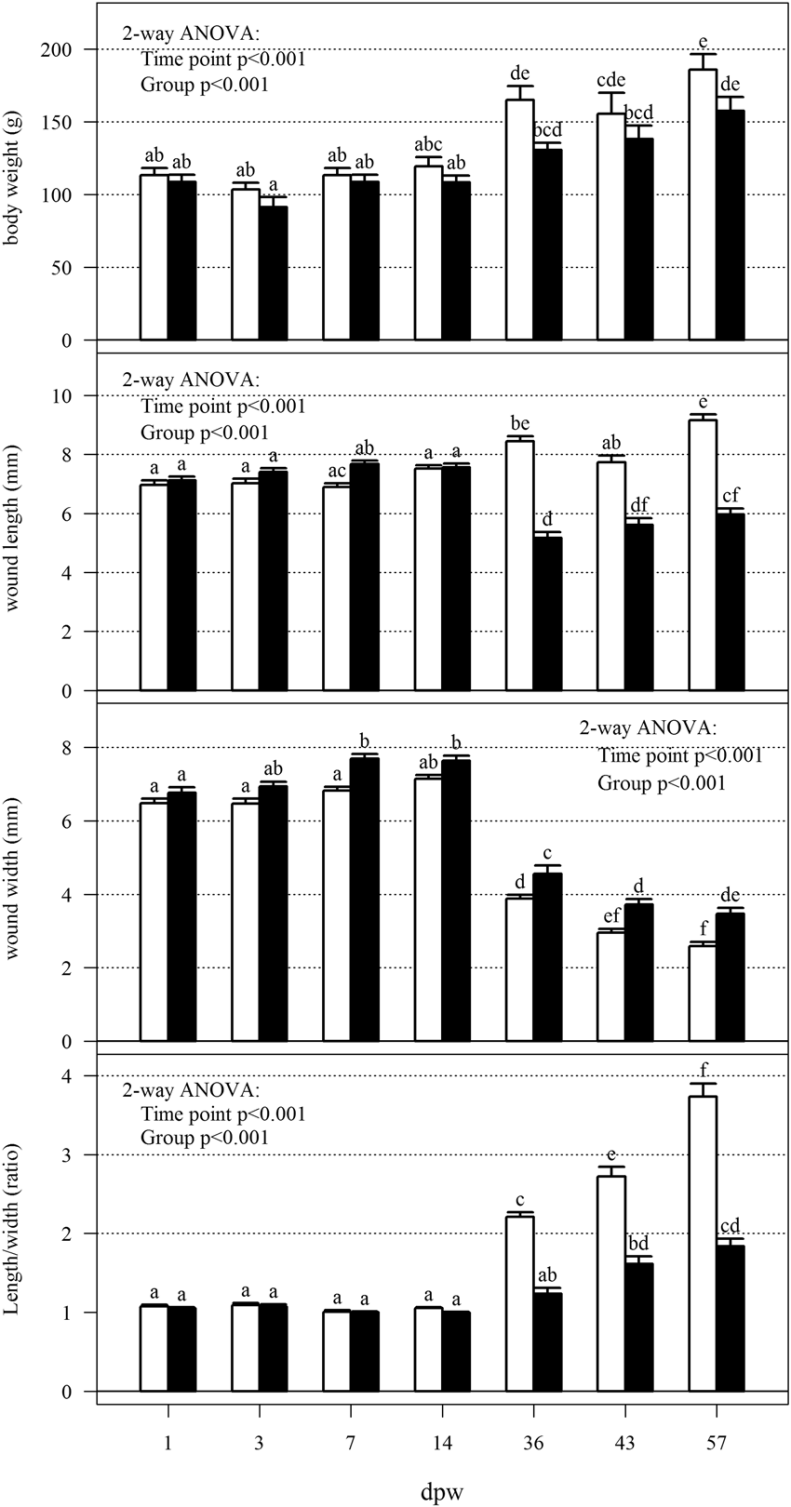
Wound measurements and body weight. Comparison of body weight, wound width, wound length and length/width ratios of the wounds. Solid bars represent the group mean and error bars SEM. 2-way ANOVA indicate significant differences between group (p < 0.001) and time point (p < 0.001) for all measurements. Lower-case letters mark differences between groups (Tukey *post-hoc* test). Groups which do not share a letter were significantly different (p < 0.05). N = 12 for treatment and time point.

**Figure 3:**
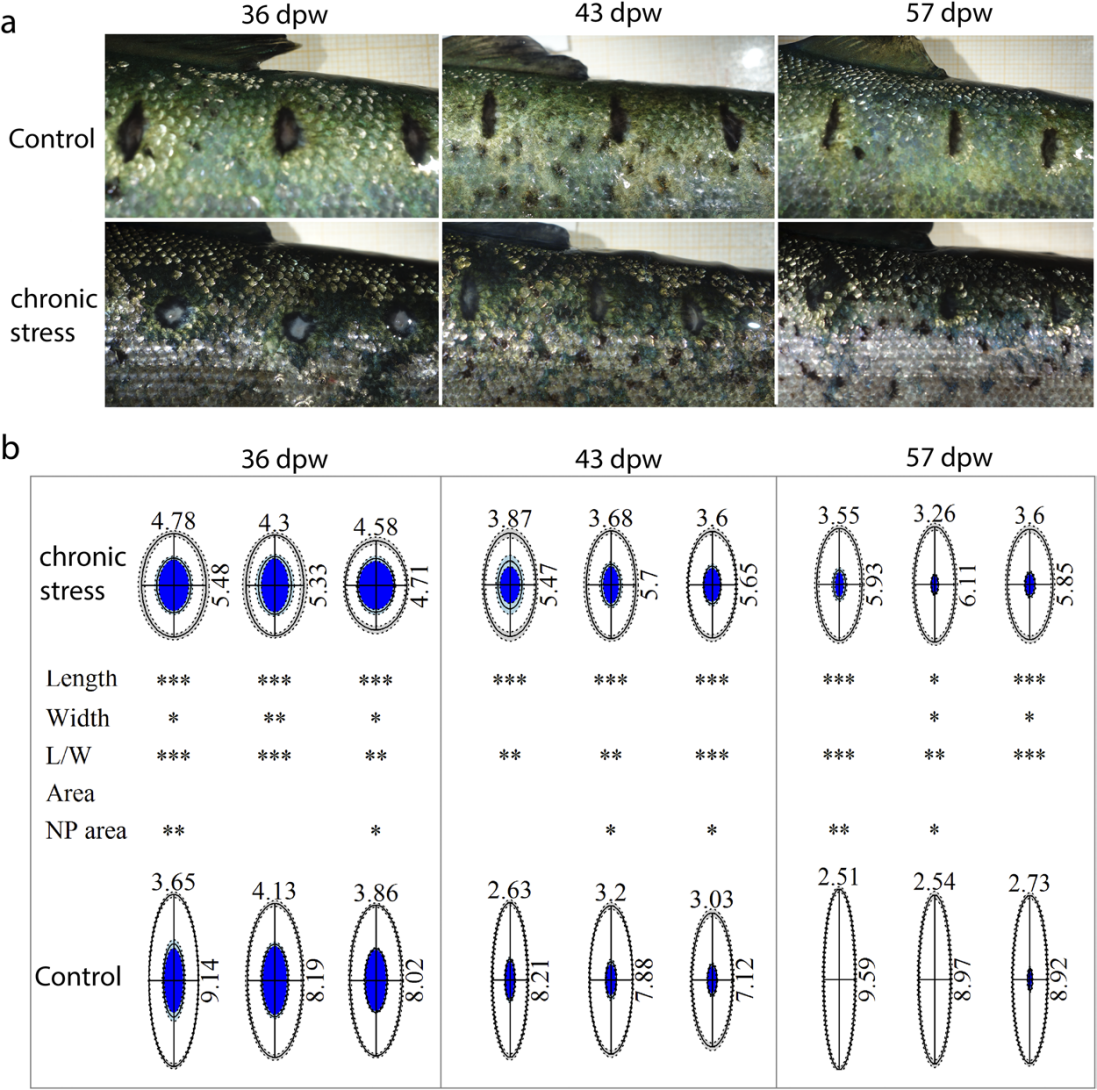
Wound morphology and wound contraction. (**a**) Representative photos of wound development at 36, 43 and 57 dpw in HFD and control treated fish. (**b**) The graphics present the total wound area as an elliptic figure. The length (mm) and width (mm) measurements are indicated in the figure. The solid lines are the mean length (L) and width (W) while the dashed lines indicate SEM. The white circle represents the whole wound area, the blue circle represents the inner non-pigmented area (NP). Significance levels of pairwise comparison of wounds in the same position and at the sampling point is indicated, stars indicate significance levels (P-value *0.05, **0.01, ***0.001) according to Kruskal-Wallis rank test. N = 12 for treatment and time point.

To investigate how HFD affected the overall wound morphology and wound contraction, the length, width, total wound area and non-pigmented inner area of the wounds was measured. The results showed that HFD altered all measured parameters, except the total wound area (Figs 2 and 3). As a measure for wound morphology, the length and width ratios (l/w) of the wounds were calculated. Wounds from the control fish had a higher (l/w) ratio (p < 0.01) compared to the wounds of HFD treated fish from 36 dpw and onward. Thus, wounds from the HFD treatment were contracting in a more circular manner compared to control wounds. Differences was also observed between the inner non-pigmented wound area, being larger at the last three sampling points in the HFD treated samples. Fish weight and wound position did not have any significant impact on wound contraction (ANOVA, p-value > 0.05, data not presented).

### Microarray

To better understand the molecular processes behind the alterations in wound contraction, the transcriptomic response in the wounds was measured with a 15k oligonucleotide microarray. The effect of HFD was strongest at 3 and 7 dpw, with 254 differentially expressed genes (DEG) at 3 dpw, and 206 DEG at 7 dpw (Table 1). It should be mentioned that the general transcriptomic profile in the two treatments were similar. None of the DEG changed direction, up vs. down, because of HFD. The effect of HFD was therefore only on the magnitude of transcription.

**Table 1:** High fish density changes the transcriptional response in the wounds.

DPW	0	1	3	7	14	36	43	57
DEG	14	58	254	206	140	46	72	41

The table shows total number of differentially expressed genes (DEG), HFD – control, at 0–57 days post wounding (dpw). Day 0 represents intact skin. Genes having a p < 0.05 and log_2_FC > ± 0.8 (fold change 1.75) were considered significantly different from each other.

**Figure 4:**
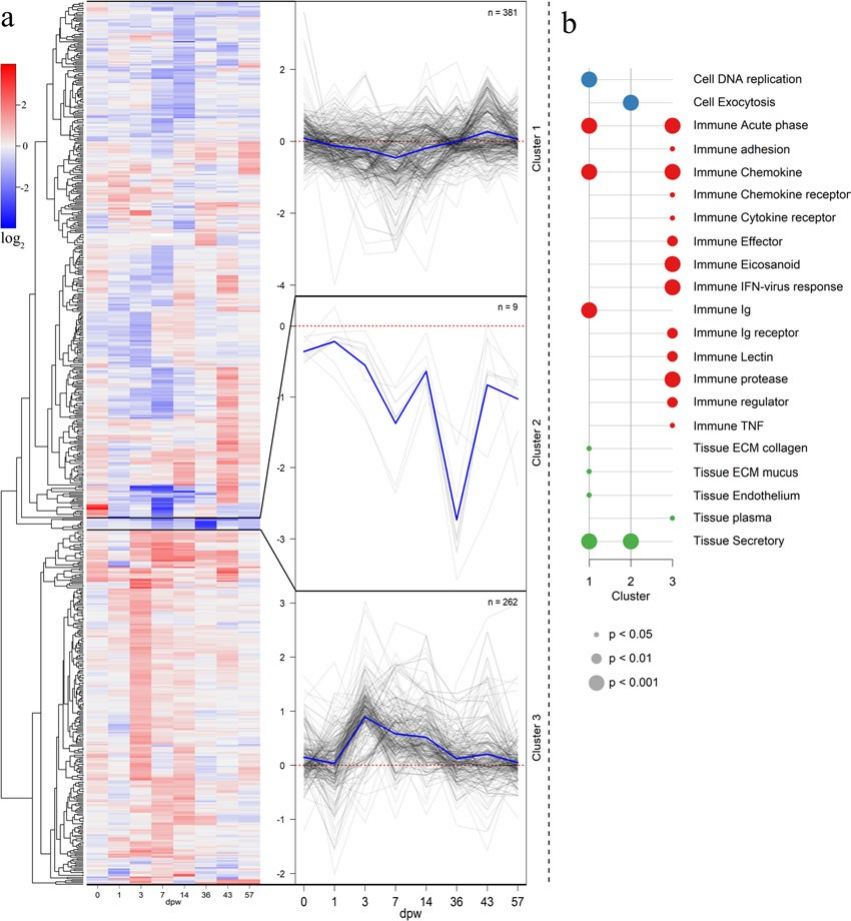
Transcriptomic responses to HFD. (**a**) The heat map shows the transcription profile of 652 differentially expressed genes (DEG). The colors represent log_2_FC of HFD vs. control samples. Red color indicates enhanced transcription in HFD samples whereas blue color represents repressed transcription. To the right, three clusters were drawn based on the transcriptional profiles of the DEG. The transcription profiles for each gene within the respective cluster is presented as a thin grey line, blue lines represent the average within the cluster. (**b**) The plot shows the enrichment results for functional categories found within each of the three clusters, cluster 1, 2 and 3 respectively. The sizes of the dots indicate the Fisher-test p-values (0.05, 0.01 and 0.001), and the color indicates enrichment category; blue for “cell”, red for “immune” and green for “tissue”. N = 5 for treatment and time point.

Clustering of DEG with known roles (N = 652) was performed for functional interrogation of transcriptomic differences between the two treatments (Fig. 4a). The majority of genes in the first cluster were downregulated by HFD the first two weeks of the experiment. Most of these genes were involved in secretion, DNA replication and immunity (acute phase, chemokines and immunoglobulins), genes encoding components of mucus and collagens were also found in this cluster (Fig. 4b). Genes in the second cluster were in general downregulated by HFD during the whole experimental period. Genes in this cluster were involved in secretion and exocytosis. Genes within the third cluster were in general enhanced by HFD. Most of these genes were involved in immune functions such as eicosanoids, lectins, proteases, cytokines and chemokines. Overall, the cluster analyzes indicate that HFD in general enhance the immune responses while tissue regeneration was repressed during the first two weeks after wounding.

### Inflammation

**Figure 5:**
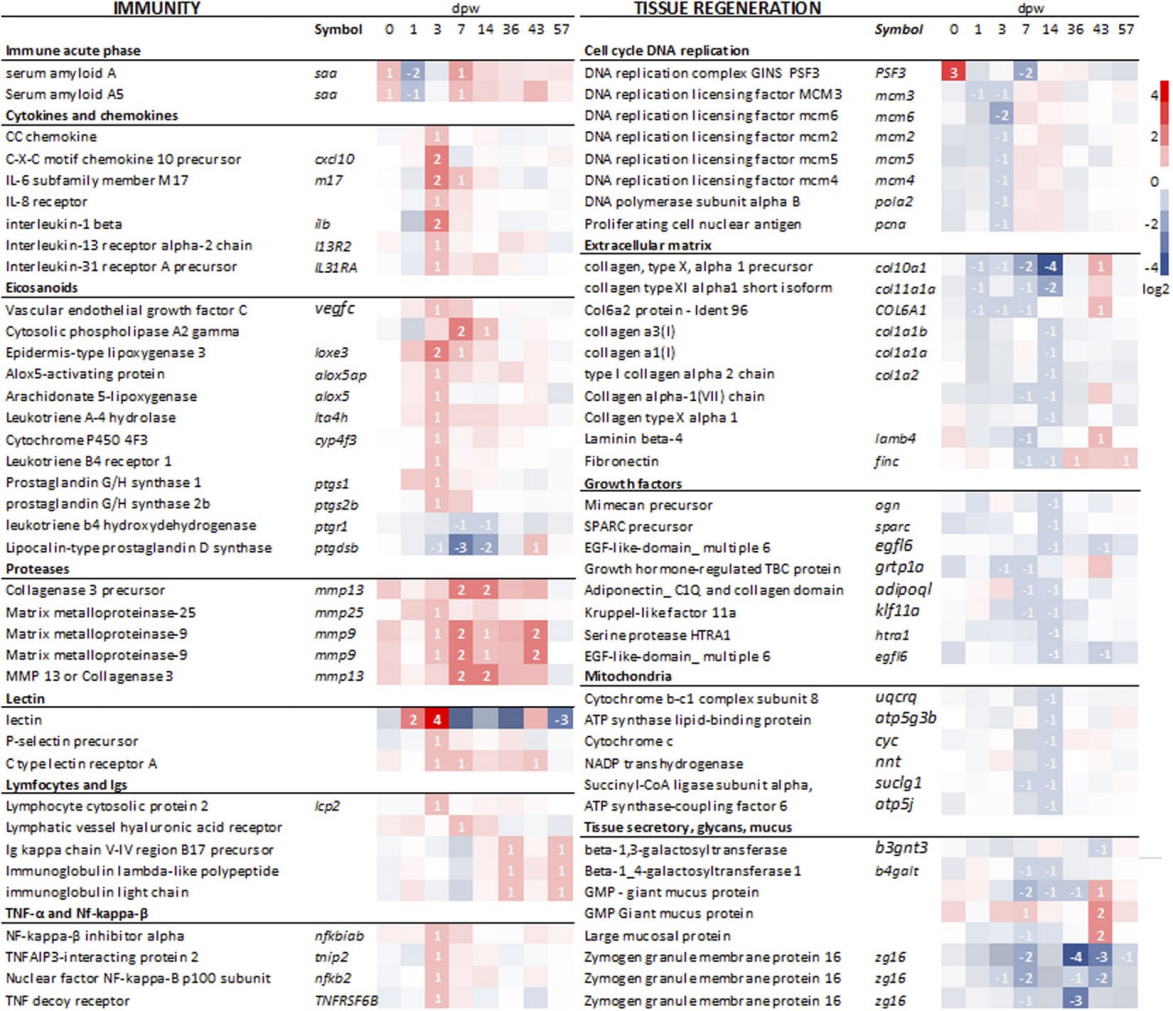
Selected genes regulated by HFD The plot to the left shows an overview of selected genes with immune functions, the right plot shows selected genes involved in tissue repair. Red color indicates up-regulation and blue color down-regulation relative to control samples. Genes with a p-value < 0.05 and a Log_2_FC > 0.8 were considered significantly different from each other and are indicated by their Log_2_FC. N = 5 for treatment and time point.

As suggested by the cluster analysis, a wide range of immune genes were up-regulated in the HFD group, including eicosanoids, lectins, proteases, cytokines and chemokines (Fig. 5). Most of the inflammatory genes showed a transient response to HFD, with enhanced transcription levels at 3 and/or 7 dpw. This included multiple genes involved in the metabolic pathway of leukotriene B4 (*Cytosolic phospholipase A2*, *Prostaglandin G/H synthase*, arac*hidonate 5 lipoxygenase, lipoxygenase 3, leukotriene A4 hydrolase, cytochrome P450 4F3*, *leukotriene B4 receptor 1*). Leukotriene B4 is known to be a major product of activated neutrophils and macrophages, with the ability to recruit and activate a range of immune effector cells^22^.

Transcription of several proteases was enhanced by HFD at multiple time points. The *Matrix metalloproteinases* 9 and 13 (*mmp9* and 13) showed a four-fold higher difference during the first two weeks of the experiment (Fig. 5). These proteases were the immune genes that were longest and strongest induced by the HFD treatment. Matrix metalloproteinases are secreted both by keratocytes and macrophages, and they are essential components of several wound healing processes (Schultz *et al*., 2005). Since both *mmp9, mmp13* and *leukotriene b4* are produced by leukocytes, these results may indicate stronger recruitment of inflammatory cells in the HFD wounds. In this context, it is relevant to mention that the transition from inflammation to tissue regeneration is dependent on matrix metalloproteinases activity^23–25^. Enhanced proteolytic activity, in particular *mmp9* and *mmp13*, is reported as a key factor causing chronic and delayed wound healing in mammals^26,27^.

### Tissue repair

Transcription of genes involved in tissue repair was temporarily repressed by HFD. Several genes involved in DNA replication were repressed at 3 dpw (Fig. 5), including *proliferating cell nuclear antigen* (*PCNA*). At 1, 3 and 7 dpw, several collagens were down-regulated by HFD and this effect was further enhanced at 14 dpw with downregulation of multiple collagens, growth factors and mitochondrial genes. This transcriptional response shows several similarities with our previous study where a panel of collagen genes and growth factors were down-regulated in the skin of Atlantic salmon with cortisol injections^28^.

**Figure 6:**
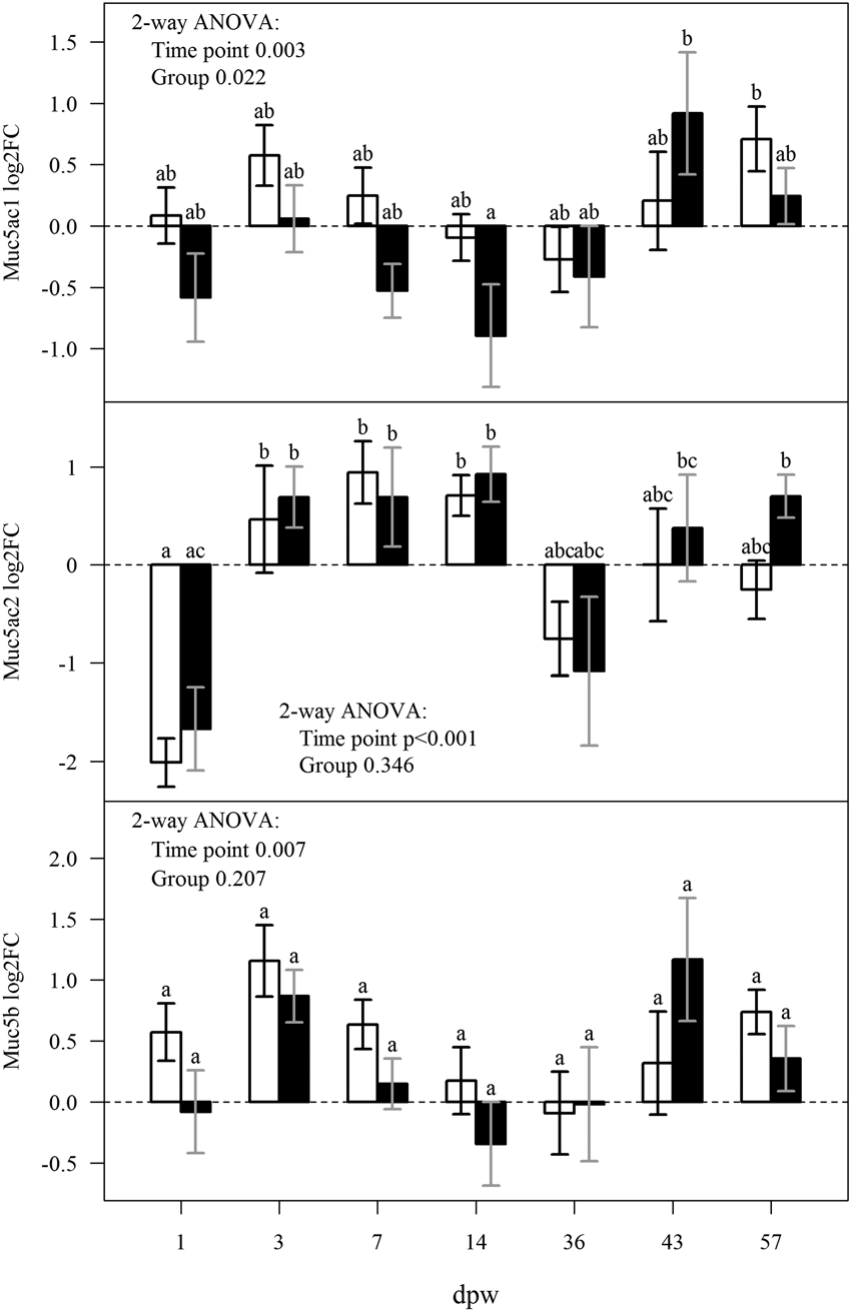
HFD alters mucin transcription. The bars show the mean transcription levels of three measured mucin genes and error bars ± SEM. Fold changes are relative to mean values of intact skin (n = 10) and log_2_ transformed. The HFD treatment is represented by black bars and the control treatment by white bars. Lower-case letters mark differences between groups (two-way ANOVA, Tukey *post-hoc* test). Groups which do not share a letter were significantly different (p < 0.05). N = 5 for treatment and time point.

Several genes involved in secretory functions and mucus responses were also dampened by HFD. The genes being strongest down-regulated by HFD was several transcripts of *zymogen granule membrane protein 16*. In mammals, this protein is found in mucous-secreting cells of the digestive system^29^, and therefore likely to be involved in mucous secretion in fish skin. Also glycosyltransferases, which are involved in glycosylation of mucins and two *giant mucus proteins* were down-regulated at 7 dpw in the HFD treatment. Further, our results indicate that transcription of *muc5ac.1* was affected by treatment (p < 0.05), with lower transcription in the HFD treated fish at six out of seven time points (Fig. 6). Transcription of *muc5b* and *muc5ac.2* changed during the healing process, but transcription was not affected by treatment. These results are contradictive to our previous findings where high biomass led to increased mucin transcription in the skin^4,30^. However, acute handling stress had the opposite effect reducing mucin transcription^30^. Synthesis and secretion of large amounts of high molecular weight proteins with heavy glycosylation represents a significant metabolic commitment of the cell^31,32^. Hence, the observed reduction in transcripts related to mucus production in the HFD treatment may be an allostatic response to a challenging environment.

### Histology

In concordance with the wound measurements and the transcriptional results, histological analysis of tissue samples further confirmed that HFD resulted in delayed epidermal repair, scale mineralization and formation of dermis.

### Epidermal repair

**Figure 7:**
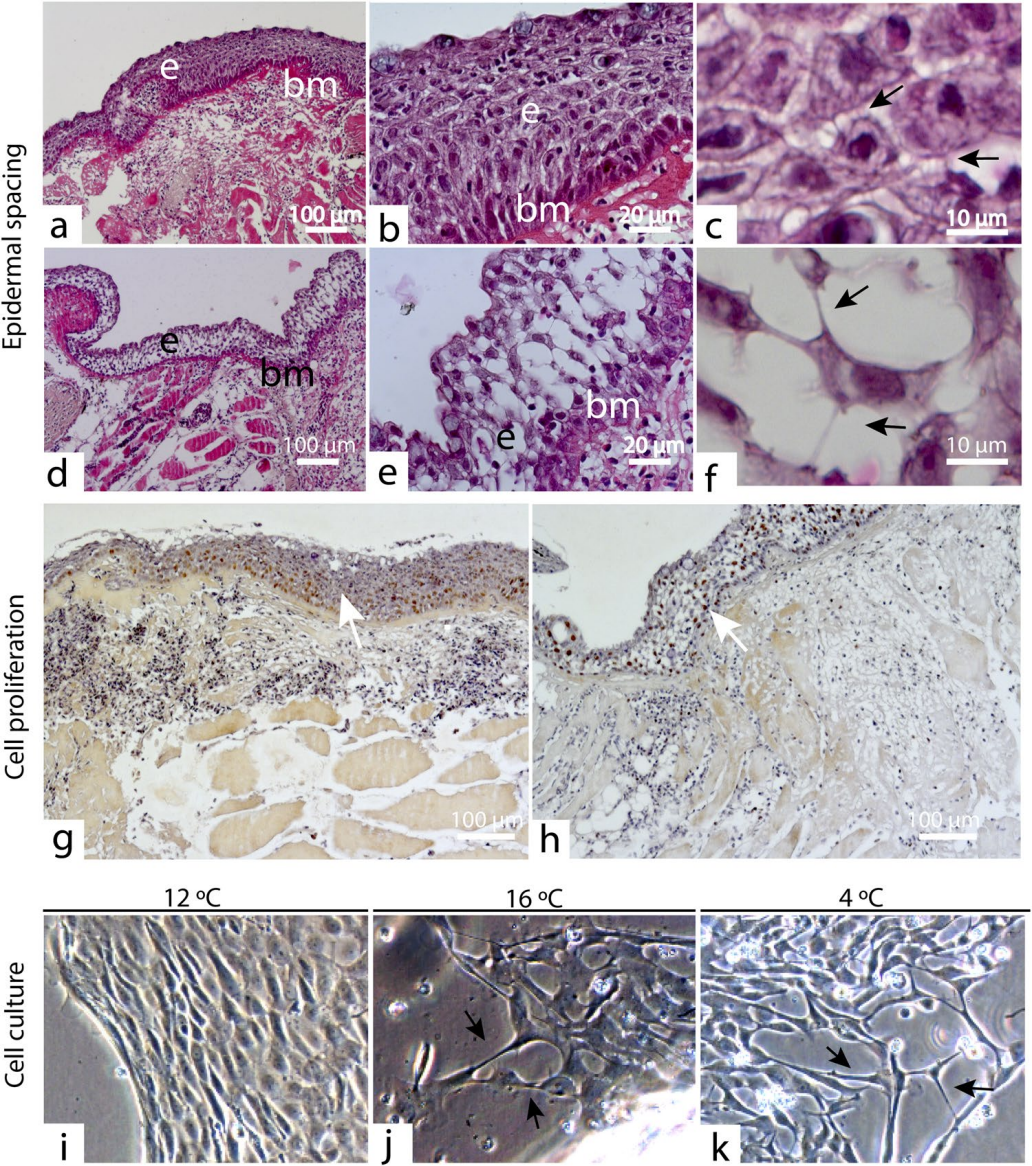
HFD and temperature-stress induces epidermal spacing. (**a**–**c**) Dense epidermal layer at 3 dpw in the control treatment. The same picture is displayed at three different magnifications (10, 40 and 60×). Note the extension of small pseudopods in c. (**d**–**f**) The epidermal layer at 3 dpw in the HFD treatment. The same picture is displayed at three different magnifications (10, 40 and 60×). Note the long extended pseudopods in f. (**g**) Plenty of dividing cells (PCNA+) were found in the epidermal layer of control fish. (**h**) PCNA+ cells in the epidermal layer in the HFD treatment. (**i**–**k**) Primary cell cultures of fish keratocytes incubated at three different temperatures 4 °C, 12 °C and 16 °C. Symbols; epidermis (**e**), basement membrane (bm), scale (sc), black arrows (pseudopods), white arrows (PCNA + cells). Hematoxylin and eosin stained tissue section (**a**–**f**), N = 5 control samples, N = 6 HFD samples. Immunohistochemistry with PCNA, nuclei of dividing cells stain brown (**g**,**h**), N = 3 for both treatments. Cell culture experiment, N = 9.

We observed a clear effect of the treatment on the epidermal layer at 3 dpw (Fig. 7). Severe epidermal spacing was observed in five out of six HFD samples, compared to one in five control samples (Supplementary File 1). The epidermal spaces were caused by keratocytes with extended pseudopods, resulting in extracellular spaces in the epidermis (Fig. 7d–f). Immunohistochemistry with PCNA further showed that cell proliferation at 3 dpw was mainly located in the epidermal layer (Fig. 7g,h). The observations also suggests less proliferative activity in the epidermis in the HFD treatment, a finding also supported by the transcriptional results (Fig. 5). The observed epithelial spacing at 3 dpw could be a side effect of reduced epidermal cell proliferation. Another factor, which may have contributed to the reduced epidermal repair, could be enhanced protease activity in the wounds. In murine models, excessive protease activity provoked type-IV collagen degradation and resulted in delayed epithelial migration^26^.

Similar observations with epidermal spacing have also been reported in Atlantic salmon with 5 mm punch biopsy wounds reared at low temperatures (4 °C)^16^. In order to further investigate the relationship between the environment and the morphology of the keratocytes, a cell culture experiment with primary keratocytes was performed. The stress factor in this experiment were low (4 °C) and high (16 °C) temperatures. The results showed that keratocytes reared at both high and low temperatures had longer pseudopods compared to the control cells (12 °C) (Fig. 5g–i). This morphology was similar to the epidermal cells at 3 dpw in the HFD treated fish (Fig. 5d–f). It is known that the mitotic rate of the keratocytes is temperature dependent^11^, however it is unclear if the observed epidermal spacing emerged due to reduced cell proliferation in the wounds or as a response to the environment.

### Mucus response

**Figure 8:**
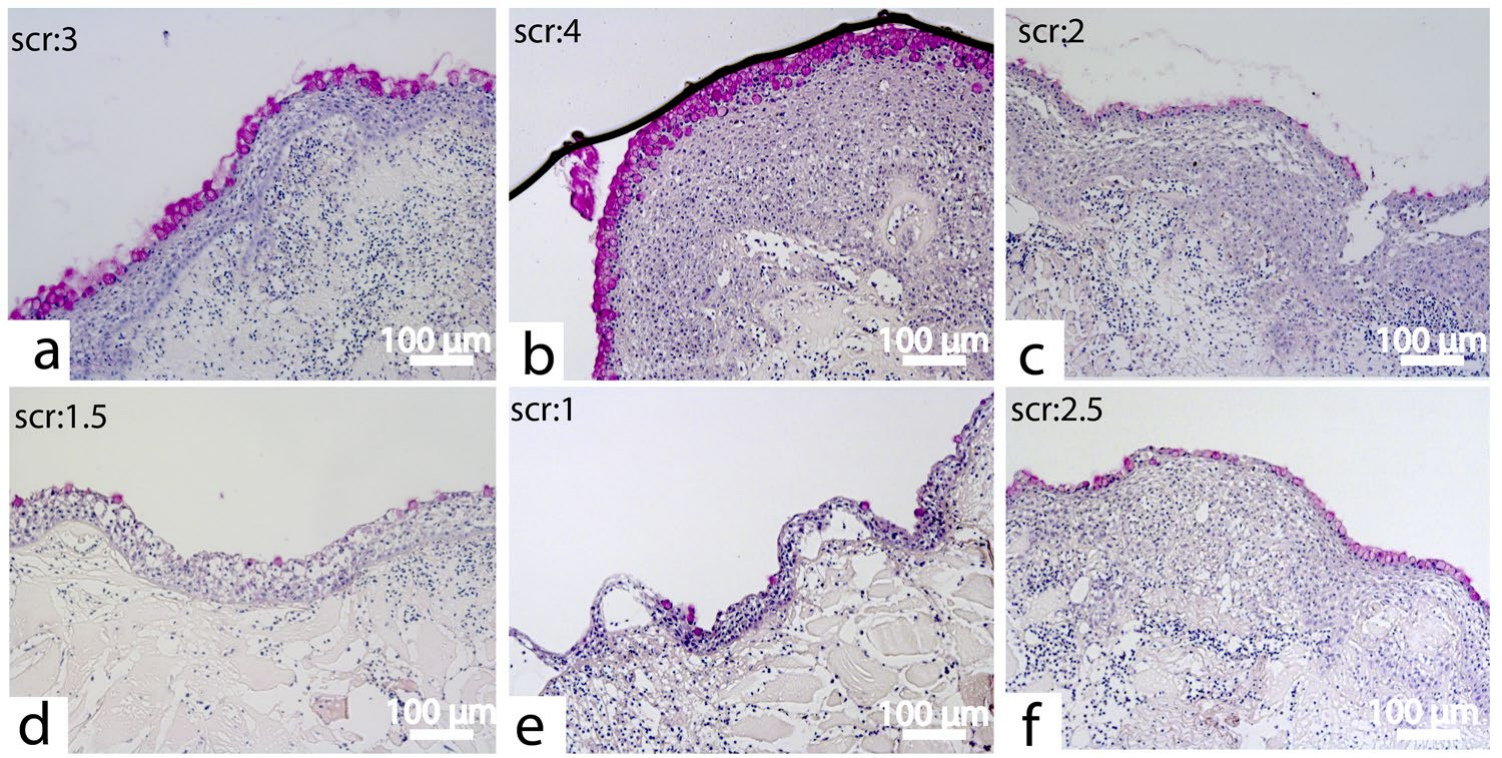
Mucus response in the HFD treatment 7 days post wounding. (**a**–**c**) The mucus response in the control samples at 7 dpw. (**d**–**f**) The mucus response in the HFD treatment at 7 dpw. Each photo represents one individual and the mean mucus score is indicated. The tissue sections (5 µm) were stained with periodic acid-Shiff to detect mucus and mucous cells (pink color). N = 5 for each treatment.

A clear difference in the mucus response between control and HFD treatment was observed at 7 dpw. At this time point there were less mucus and mucous cells on HFD samples (Fig. 8). The average mucus score of the wounds from HFD treatment was 1.7 (SEM ± 0.34) while the average score of the control samples were 3 (SEM ± 0.31) (Supplementary File 1). Combined with the transcriptional results with down-regulation of mucins, zymogens, transferases and giant mucus proteins (Figs 5 and 6), these findings strongly suggest that HFD dampens the mucus response at 7 dpw. The poor mucus response may be an indirect effect of the delayed organization of the epidermal layer at 3 dpw (Fig. 7). The rapid re-epithelialization process, followed by formation of a neo-epidermis and a mucus plug is believed to be essential in order to protect the healing wound^16,33^. Here we show that increased epidermal spacing and reduced mucus production is an early consequence of HFD, which in turn may affect the ability of the wound to withstand secondary infections.

### Scale formation

**Figure 9:**
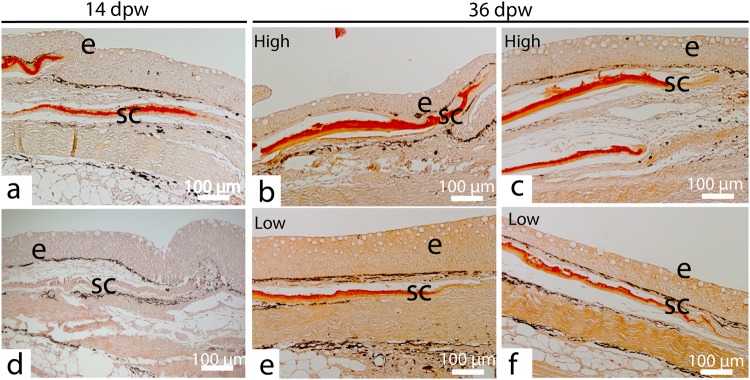
HFD delays scale mineralization. (**a**) Control sample at 14 days post wounding (dpw) with mineralized matrix (red color) in a newly formed scale. (**b**,**c**) Mineralized collagen plate from two different individuals, control samples at 36 dpw. Degree of mineralization is indicated as high or low. (**d**) HFD treated sample with newly formed scale. Note that the matrix does not stain red with Alizarin red. (**e**,**f**) Mineralized collagen plate from two different individuals, HFD samples at 36 dpw. 5 µm tissue sections stained with Alizarin red, N = 6 at 14 dpw, N = 3 at 36 dpw for each treatment.

At 14 dpw formation of new scales at the wound margins was observed in all the analyzed samples (Fig. 9a,d). In the HFD treatment, none of the scales contained mineralized matrix. In contrast, three out of five control samples contained scales with mineralized matrix. This trend continued at 36 dpw with weaker staining, on both right and left scale, in the HFD treated fish (Fig. 9b,c,e,f). As fish scales consist of an upper mineralized layer of hydroxyapatite and a lower fibrous layer of un-mineralized matrix and collagen fibers^34^, delays in collagen transcription as shown by the array results (Fig. 5), may cause a delay in scale formation and mineralization. In the European eel (*Anguilla anguilla*), vertebral bone demineralization has been observed after chronic cortisol treatment^35^. Further, Atlantic salmon vertebra is less mineralized when exposed to increased temperature stress, such as 16 °C compared to 12 °C^36^. The same has also been shown for salmon mesenchymal stem cells differentiating to bone cells *in vitro*^37^. Overall, HFD may have a negative effect on scale development and mineralization, which may impair the mechanical barrier of the fish.

### Fibrous repair and pigmentation

**Figure 10:**
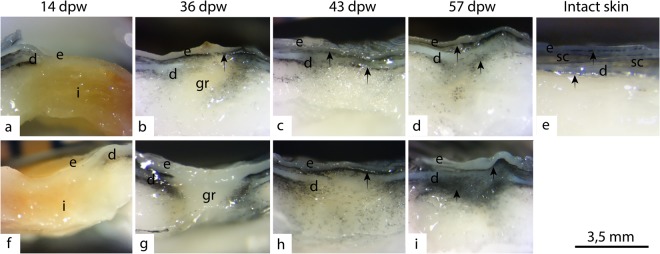
Delayed formation of pigmentation and dense connective tissue. Representative photos of unstained tissue samples from control and HFD treatment, 14-57 days post wounding. (**a**–**d**) Healing wounds from control fish. (**f**–**i**) Healing wounds from HFD treated fish. Epidermis (**e**), dermis (**d**), inflammation (**i**), granulation tissue (gr). Arrows point at pigmentation beneath the epidermal layer, and beneath the dense connective tissue. Stereoscope pictures (40×), N = 6 for each treatment and time point.

Fibrous repair and restoration of skin pigmentation was delayed by HFD (Fig. 10). In normal fish skin, the pigment cells are localized below the epidermal and dermal layer (Fig. 10). At 36 dpw, four out of six control samples and only one out of six HFD samples had a layer of melanocytes below the epidermal layer (Fig. 10b,g). At 43 dpw, five out of six control samples had pigment cells organized in two layers, under the epidermal and dermal layer, while none of the HFD samples had this organization (Fig. 10c,h). These data support our findings that wounds from HFD treated fish retains a bigger non-pigmented area in the wound center (Fig. 3). Furthermore, the dermal layer looked more organized in the control samples, thus the pigment cells appear to follow the formation of connective tissue. At 57 dpw all samples had melanocytes organized beneath the epidermal and dermal layer, suggesting that tissue repair in the HFD treated fish was catching up on the control (Fig. 10d,i). The transcriptomic results also support this finding, with higher collagen transcription in wounds from HFD treated fish at 43 dpw (Fig. 5).

Cortisol treatment in Atlantic salmon and zebrafish (*Danio rerio*)^10,12^, and chronic stress situations in mice and humans is associated with reduce dermal repair and delayed wound contraction^38–43^. Given these results presented in this article, we believe that chronic stress and associated physiological responses is the best explanation for the delayed wound healing in the HFD treatment.

## Conclusion

**Figure 11:**
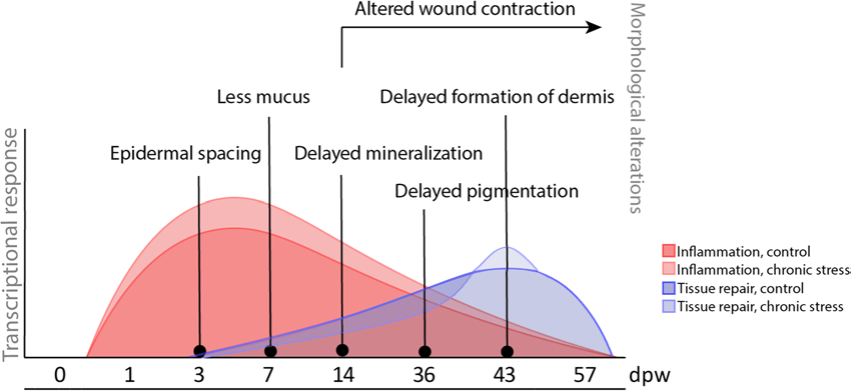
Summary of events that are altered by HFD in the healing wounds of Atlantic salmon. Inflammation and tissue repair were the two dominating transcriptional responses to wounding. In general HFD enhanced transcription of genes related to diverse inflammatory responses, while tissue repair was repressed at most time points. This resulted in several transient morphological changes in the wound and permanent alterations in wound contraction.

The results presented in this article show that HFD interferes with the wound healing capacity in Atlantic salmon. At the transcriptional level, the HFD treatment enhances inflammatory reactions in the wound while repressing tissue repair (cell proliferation, tissue secretion, and collagen production) (Fig. 11). The observed alterations in gene transcripts had a lag time, manifesting themselves at later time points on the morphological appearance of the wounds. These morphological differences included poor epidermal organization, delayed scale mineralization, delayed the formation of fibrous tissue and altered wound contraction. Combined, our findings suggest that HFD interferes with the wound healing capacity of the fish resulting in delayed epidermal and dermal repair.

## Materials and Methods

### Fish stock, rearing conditions and sampling procedure

This study was carried out at the Industrial Laboratory (ILAB, Bergen Norway) between November 4^th^ 2014 to January 30^th^ 2015. Smolts (mean size 80 g) were distributed randomly in two 500 L tanks. The fish were stocked at 20 kg/m^3^ (N = 125) in the control treatment and at 100 kg/m^3^ (N = 625) in the HFD treatment. From the 4^th^ to the 6^th^ of November the fresh water in each tank was gradually replaced with seawater. The specific water flow was adjusted to 5 L/min in the control tank and 25 L/min in the HFD tank, corresponding to 0,5 L/kg/min at the start of the experiment. Water velocity (10 cm/sec) in each tank was kept stable and equal by adjusting the angle on the inlet water pipe. The oxygen level in the outlet water was kept higher than 80% saturation by automatic oxygenation of the water in the header tanks (Oxyguard Commander). Both temperature (ranging from 9.4–10 °C) and oxygen saturation were measured once daily (YSI 550, Xylem Inc., Yellow Springs, USA). Following transfer to full strength seawater the fish were exposed to a 12 hours light and 12 hours dark regime. The fish were fed a commercial dry diet (EWOS, size 2–3 mm, Oslo, Norway) in 10% excess throughout the study. The main experimental period lasted from 28^th^ of November 2014 until 30^th^ of January 2015. The biomass was not adjusted in order to avoid adding additional stressors to the experiment. As a result the fish growth were greater than the biomass sampled in the HFD treatment, causing the biomass to increase over the time course of the study. The fish density in the HFD group ranged from 116 to 146 kg/m^3^, with a mean fish density of 126 kg/m^3^. The fish growth in the control treatment did not compensate for the biomass lost at sampling, thus the fish density was reduced over time, ranging from 6 to 22 kg/m^3^ with a mean fish density of 14 kg/m^3^. Four weeks after transfer to seawater three biopsies (N = 90 per treatment), were excised with a 5 mm biopsy needle (Integra^TM^Miltex^TM^) as described by several authors^16,44^. Prior to wounding the fish were fully anesthetized with (MS22, Sigma-Aldrich). Skin samples were taken at 1,3,7,14,36,43 and 57 dpw (N = 12 for time point and treatment).

Fish for sampling were killed with an overdose of anesthetic (MS-222). Individual fish were weighed (g) and their length measured (cm). Blood was sampled with a heparinized syringe (Omnifix-F) from the caudal blood vessels and centrifuged (10 min at 4 °C and 4000 rpm). Plasma was stored at −80 °C for further analysis. Skin samples were collected from a standardized 1 cm^2^ area around each wound. Samples for gene transcriptional analyses were snap frozen in liquid nitrogen and transferred to −80 °C for storage. The skin samples were fixed in 4% Paraformaldehyde solution (Electron microscopy science) overnight and then washed in 1 x PBS (Sigma Aldrich), before stepwise dehydration to 70% ethanol and transferred to −20 °C for storage.

### *In vitro*-study, primary cell culture

In order to investigate the effect of temperature stress on keratocyte morphology, an *in-vitro* study with primary cell cultures were established. Keratocytes were cultured from scale explants, a method modified from previous work^45,46^. In brief, Atlantic salmon (N = 9, weight ~ 500 g), were killed by a blow to the head and transported from the rearing site (NIVA Research station, Solbergstrand, Norway) to Nofimas’ research facilities (Aas, Norway) in transport tanks with seawater. Single scales were picked (using forceps) and placed in 6 well tissue culture plate (Falcon Multiwell™ Becton Dickinson, NJ, U.S.A.) containing L-15 supplemented with fetal bovine serum (FBS) 10% (Sigma), 25 μg amphotericin B, 10 mL/L antibiotics (Sigma), 10 mL/L antimycotics (Sigma) and 0.01 M HEPES (Sigma). Each well contained three scales, and for each fish three plates were used. Each plate were cultured at one out of three different rearing temperatures: control (12 °C), low temperature (4 °C) and high temperature (16 °C). The temperatures were chosen based on optimum (12–14 °C) rearing temperature for Atlantic salmon as both lower and higher temperatures are associated with reduced fish growth^47,48^. After four days the cells were microscopically analyzed (Leica).

### Cortisol analysis

A direct enzyme immunoassay (EIA) was used to measure plasma cortisol^49^. Samples were added to 96 well plates coated with Rabbit anti-cortisol (Cat# 20-CR50, Fitzgerald Ind. Int’l, North Acton, MA, USA; diluted 1:30000). Color development was measured at 650 nm by an automatic plate reader (Sunrise BasicTM, software: MagellanTM V6.5, Tecan Group Ltd, Männedorf, Switzerland). Maximum binding (B0 = 150 µl EIA + 100 µl cortisol – HRP conjugate) and non-specific binding (NSB = 150 µl EIA − 100 µl cortisol – HRP conjugate) were determined. All standards were run in triplicates and samples in duplicates.

### Photography

Photographs were taken with Cyber-shot DSC-RX100 (Sony) with an internal calibration standard in each picture. The length, width, non-pigmented area and total wound area were measured with Image J (Image J. Inc), (N = 6 at 1–7 dpw and N = 12 at 14–57 dpw for each treatment).

### Histology

Skin samples for histology were embedded in paraffin, using the program 70%, 96%, and 3 × 100% et-OH, 3X xylene and 2X paraffin, total duration of 10 h (Leica TP1020). Following embedding, samples were sectioned into 5 µm sections. All the sections were stained with haematoxylin-eosin (Sigma-Aldrich) with an automatic staining machine (Leica autostainer XL). Staining with periodic acid-Schiff was done by oxidizing the sections in 0.5% periodic acid solution (Sigma-Aldrich) for 5 min, followed by Schiff reagent (Merck) for 15 min, and counterstaining in Mayer’s hematoxylin (Sigma-Aldrich). Staining for alizarin red (Sigma Aldrich) were done in a solution of 2 g Alizarin red in 100 mL dH_2_O, pH 4.3 for 2 min. Samples were dehydrated in increasing alcohol gradient (50–100%) and cleared in xylene. The slides were mounted with Fully Automated Glass Coverslipper (Leica CV5030). Staining of Proliferating Cell Nuclear Antigen (PCNA), was done with mouse anti-PCNA IgG2a (Millipore) and VECTASTAIN® Abc - HRP kit, anti-mouse IgG (Vector Laboratories) according to the manufacturer’s instructions (N = 3).

Three independent researchers analyzed the samples blind for mucus, epithelial spacing and scale mineralization, all scores may be found in Supplementary File 1. The amount of mucus present on samples were scored in a 10X magnification area on a scale from 0–4. Score values were defined as 0 (no mucous cells), 1 (less than 15 mucous cells), 2 (more than 15 mucous cells partly forming a continuous layer), 3 (one continuous layer of mucous cells), 4 (two continuous layers of mucous cells). Epidermal spacing were scored on a scale from 0–3 in a 5X magnification area, with the following scoring values: 0 (normal epidermis with no-spacing between keratocytes), 1 (little epidermal spacing), 2 (occurrence of epidermal spacing) and 3 (severe epidermal spacing). Since quantification of alizarin in sections is complicated, scoring of alizarin stained scales was defined as those with the strongest staining gained the maximum score “high”, those with less staining were classified as “low”.

### RNA extraction

Frozen skin sections with wounds were cut in half by a diagonal section, and repeated, and approximately one-fourth of the wound were transferred directly to 1 mL TRIzol (Thermo Fisher Scientific). Samples were homogenized in a Precellys®24 homogenizer. RNA was extracted from the homogenized tissues using PureLink™ Pro 96 well purification kit (Thermo Fisher Scientific) with on-column-DNase (Qiagen) digestion according to the protocol for TRIzol-homogenised samples. The concentration of extracted total RNA was measured with NanoDrop 1000 Spectrometer (Thermo Fisher Scientific) and RNA integrity was determined with Agilent 2100 Bioanalyzer with RNA Nano kits (Agilent Technologies). Samples with RNA integrity number (RIN) of 8 or higher were accepted.

### Microarray

Analyses were performed with Nofima’s Atlantic salmon DNA oligonucleotide microarray SIQ-6 (custom design, GPL16555) containing 15 K probes of genes selected by annotations and expression profiles. Microarrays were fabricated by Agilent Technologies; all reagents and equipment were purchased from the same source. All kits were used according to manufacturer’s protocol. In brief, RNA amplification and labelling with Cy3 was performed with Low Input Quick Amp Labeling Kits (200 ng of total RNA per reaction) and Gene Expression Hybridization Kits were used for fragmentation of labelled RNA and preparation of the hybridization setup. Microarrays were hybridized for 17 h in a hybridization oven at 65 °C and rotation speed of 10 rpm, washed for one minute with Gene Expression Wash Buffer I at room temperature, and one minute with Gene Expression Wash Buffer II at 37 °C. Washed slides were scanned with an Agilent SureScan Microarray scanner. Nofima’s bioinformatics package STARS^50^, was used for data processing and mining. Five replicates per group and time-point were included in analyses, and four biological replicates per group from intact skin, totally 78 arrays were used.

### qPCR

Synthesis of cDNA was performed on 500 ng RNA with SuperScript® VILO cDNA Synthesis Kit and Master Mix (Thermo Fisher Scientific). QPCR was performed in duplicates in 364 optical plates on a QuantStudio5-384w (AppliedBiosystems) in default “fast mode”. Each well had a final reaction volume of 10 μl (5 μl PowerUp™ SYBR™ Green Master Mix (AppliedBiosystems), 4 μl of 1:10 diluted cDNA and primers 0,5 μl of 10 µM forward and reverse primer). Quantification cycle (Ct) values were calculated using the second derivative method in the QuantStudio™ Design and Analysis Software v1.4.3. The efficiency of the RT qPCR reactions was estimated for all primer pairs by eight times 2-fold dilution series. RT-qPCR primers for *muc5ac.1, muc5ac.2/4* and *muc5b* and the housekeeping genes *elf1a* and *etif3* as describe by^30^. Two reference genes were evaluated for stability using the web-based comprehensive tool RefFinder which integrates the computational programs geNorm, Normfinder, BestKeeper and the comparative delta-Ct method^51^. The *etif3* and *elf1a* obtained similar stability score, while the mean value of the two genes were most stable and was therefore used in the normalization procedure. Five replicates per group and time-point were included in analyses. Relative expression ratios to mean expression levels in intact skin (N = 10, equal amounts of control and HFD) were calculated and the data was log_2_ transformed before data analysis and plotting.

### Statistics

Data analyses were performed in R (version 3.3.1, www.r-project.org). Data series were tested for normal distribution (Shapiro-Wilk normality test, R-function shapiro.test()). If the test was passed (p-value > 0.05), data were analyzed by ANOVA (R function aov()) and in case significant differences were found (p-value < 0.05), a Tukey *post-hoc* test was calculated (R function TukeyHSD()). If the normality test was not passed, Kruskal-Wallis rank tests (R function kruskal.test()) were used.

For evaluations of microarray results, the differentially expressed genes (HFD-control) were selected by the following criteria: log_2_-Expression Ratio >0.8| (1.75-fold) and p < 0.05. A complete gene list of DEG, gene identifier and their respective STARS category^52^, can be found in Supplementary File 1. The Euclidean distances were calculated, and the complete linkage clustering was drawn as a heat map. The dendrogram was pruned in order to identify 3 clearly-defined sub-clusters. For each cluster one-tailed Fisher tests for significant over-representation of functional categories (STARS) were run. Filtering, statistical analyses and plotting of results were performed in R.

### Animal statement

This study was approved by the local responsible laboratory animal science specialist under the surveillance of the Norwegian Animal Research Authority (NARA) and registered by the national ethics committee (the Norwegian Food Safety Authority, ID7058). The methods were carried out in accordance with the relevant guidelines and regulations.

## Electronic supplementary material

Supplementary file 1

## Data Availability

Data were submitted to Gene Expression Omnibus (GSE122142).
